# Emerging Insights into Liver X Receptor α in the Tumorigenesis and Therapeutics of Human Cancers

**DOI:** 10.3390/biom13081184

**Published:** 2023-07-28

**Authors:** Ning Han, Man Yuan, Libo Yan, Hong Tang

**Affiliations:** 1 Center of Infectious Diseases, West China Hospital of Sichuan University, Chengdu 610041, China; 2 Division of Infectious Diseases, State Key Laboratory of Biotherapy, Sichuan University, Chengdu 610041, China

**Keywords:** liver X receptor α, tumorigenesis, therapy, cancer

## Abstract

Liver X receptor α (LXRα), a member of the nuclear receptor superfamily, is identified as a protein activated by ligands that interacts with the promoters of specific genes. It regulates cholesterol, bile acid, and lipid metabolism in normal physiological processes, and it participates in the development of some related diseases. However, many studies have demonstrated that LXRα is also involved in regulating numerous human malignancies. Aberrant LXRα expression is emerging as a fundamental and pivotal factor in cancer cell proliferation, invasion, apoptosis, and metastasis. Herein, we outline the expression levels of LXRα between tumor tissues and normal tissues via the Oncomine and Tumor Immune Estimation Resource (TIMER) 2.0 databases; summarize emerging insights into the roles of LXRα in the development, progression, and treatment of different human cancers and their diversified mechanisms; and highlight that LXRα can be a biomarker and therapeutic target in diverse cancers.

## 1. Introduction

Liver X receptor α (LXRα), a nuclear receptor superfamily member, can recruit ligands and bind to particular DNA sequences in target gene promoters [1,2]. As one of the critical regulators, LXRα has been widely correlated with certain specific genes in metabolism processes, especially cholesterol metabolism [3]. Many studies have suggested that LXRα may exert distinct functions in different tissue-specific environments. LXRα-modulated distinct downstream target genes can engender these varied roles. LXRα may be involved in various signaling pathways by repressing or inducing the expression of these genes to maintain normal physiological activity, such as the ATP-binding cassette transporter A1 (ABCA1) and Niemann–Pick type C1 [4,5]. Moreover, the high expression of LXRα in macrophages suggests that LXRα is also an essential factor for inflammation and immunity homeostasis by regulating the expression of many inflammation-associated genes through various pathways [6]. For example, LXRα can induce the expression of interferon γ and control the immunity process via macrophage phagocytosis [7]. LXRα dysfunction is present in the pathogenesis of different diseases. For example, LXRα might contribute to antiatherosclerosis by ameliorating the function of macrophages, which includes encouraging M2 polarization, reducing the release of pro-inflammatory cytokines, and increasing the release of protective molecules [8]. In addition, some studies have suggested that LXRα could contribute to the pathogenesis of liver steatosis [9], neuroinflammatory disease [10], and cardiometabolic disease [11]. In addition to these known functions, there is increasing evidence that LXRα is associated with many cancers.

In recent years, an increasing number of studies in vitro and in vivo have shown that LXRα may be an important factor in the proliferation, apoptosis, invasion, and migration of cancer cells [12]. The abnormal expression of LXRα can be observed in many cancers and has a strong relationship with the clinical outcome. Thus, LXRα may function as a diagnostic and prognostic biomarker, and it may potentially be a target for cancer therapy. However, the expression of LXRα in different cancers is controversial, the mechanism of LXRα in tumor pathophysiology is relatively complex, and the potential clinical value of LXRα in tumor therapy is uncertain. In this review, we show the expression levels of LXRα between tumor tissues and normal tissues via the Oncomine and the Tumor Immune Estimation Resource (TIMER) databases, focus on the role and mechanism of LXRα in various cancers, and discuss the conceivable performance of LXRα in cancer treatments.

## 2. The Physiological Function of LXRs and LXRα

As early as the 1990s, LXRs had already been cloned from mice. Two isoforms were identified, named LXRα and LXRβ because of their high expression in the liver [13,14]. They are also known as nuclear receptor subfamily 1 group H member 3 (NR1H3) and nuclear receptor subfamily 1 group H member 2 (NR1H2), respectively [15]. LXRs have the typical nuclear receptor structures, which are mainly composed of four domains: the ligand-independent activation functional domain (AF-1) at the N-terminus; the DNA-binding domain (DBD) with two zinc finger motifs; the hydrophobic ligand-binding domain (LBD), which identifies specific ligands and binds to coactivators; and the ligand-dependent activation functional domain (AF-2) at the C-terminal region [16]. LXRα is encoded by the NR1H3 gene located on chromosome 11p11.2, while the NR1H2 gene on chromosome 19q13.3 can encode LXRβ [17]. LXRα and LXRβ consist of 447 amino acids and 460 amino acids, respectively, and more than 78% of their sequences are identical, mainly located in the DBD and LBD parts [15,18]. However, their expression patterns are different in the body. LXRα is mainly expressed in cells, tissues, and organs with active cholesterol and lipid metabolisms, such as the liver, intestine, kidney, spleen, lung, adipose tissue, and macrophages, while LXRβ is expressed in almost all tissues and organs [19]. Although current studies suggest that both LXRα and LXRβ are involved in lipid metabolism, there are still some differences in their mechanisms. The current study found that LXRα makes a greater contribution to the regulation of cholesterol efflux transport pathways, while LXRβ plays a greater role in lipid metabolism [20,21].

**Figure 1:**
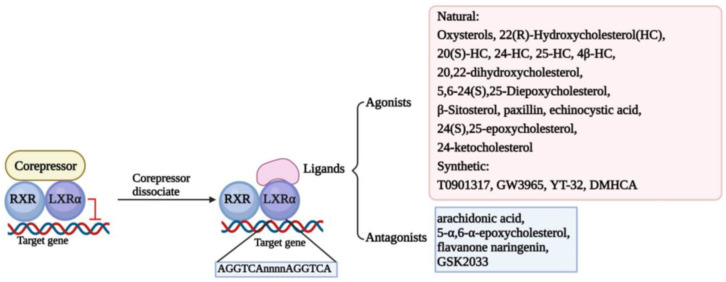
Classical mechanism and multifarious ligands of LXRα. The classical transcriptional mechanism of LXRα is binding to RXR formatting heterodimer. When the LXRα/RXR interacts with corepressors, LXRα/RXR dimer activation is inhibited, and transcription of the target genes is suspended. Once the corepressor is dissociated, the ligands could activate the function of LXRα/RXR to interact with LXRE (AGGTCAnnnnAGGTCA) located in the promoter of target genes and regulate the transcription of target genes. Some ligands are depicted here, including agonists and antagonists.

Structure determines function. The classic structure of nuclear receptors contributes to their interaction with ligands and the DNA sequence of the target gene, named LXR response elements (LXREs), finally modulating the transcription of the target gene [22]. In the screening of retinal X receptor (RXR)-binding ligands, it was found that LXRs can bind to it and form heterodimers to participate in retinol signal transduction [23]. In the absence of ligands, LXR/RXR heterodimers form complexes with classic nuclear receptor corepressors to inhibit the transcriptional activity of target genes. However, in the presence of ligands, LXR/RXR heterodimers release corepressors and recruit coactivators to transactivate the expression of target genes [16]. Thus, there is no doubt that the ligand plays an essential role in activating LXRs. The ligands of LXRs are mainly divided into two main categories, agonists and antagonists, which include natural substances and synthetic ligands [24,25]. We have summarized the ligands in Figure 1.

Although a large number of studies have shown that the classic DNA sequence of LXREs is usually “AGGTCAnnnnAGGTCA”, some studies have also demonstrated that LXRs can share binding sites with other nuclear receptors or transcription factors in some regions of chromatin, such as tumor necrosis factor-α/nuclear factor-κB [16]. Furthermore, it has been found that LXRα binds specifically and preferentially to overlapping cAMP response elements and negative response elements (CNREs) in a monomeric form, increasing the expression levels of renin. They also found that LXRα specifically binds to CNREs in the c-myc promoter, thereby increasing c-myc transcription, suggesting that LXRα may act as a cAMP-responsive transcriptional regulator to regulate gene expression [26,27]. In addition, LXRs can also be regulated by certain transcription factors or non-coding RNAs to exert antitumor effects. For instance, it was found that the transcription factor KLF4 significantly contributes to the maintenance of vascular homeostasis by regulating the expression of LXR [28]. In addition, miR-552-3p also regulates LXRα transcription by binding to the region of AGGTCA [29]. In conclusion, LXRα may regulate downstream target genes or be regulated by upstream factors, and its activation or not may affect normal cellular activities and may be involved in tumorigenic mechanisms.

## 3. The Expression of LXRα in Different Tumor Tissues

**Figure 2:**
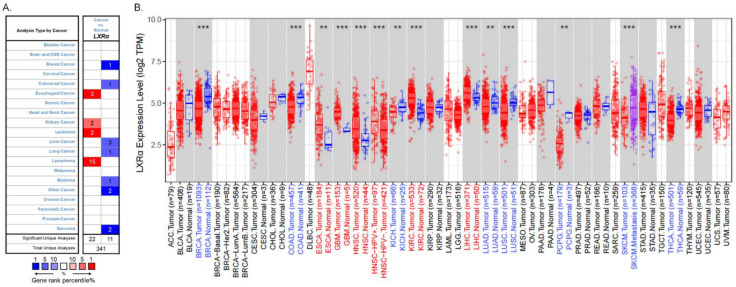
LXRα expression levels in different types of human cancers: (**A**) increased or decreased expression of LXRα in different types of cancer tissues compared with normal tissues in the Oncomine database; (**B**) human LXRα expression levels in different cancers were assessed using the TIMER 2.0 database (** *p* < 0.01, and *** *p* < 0.001).

LXRs are essential in cholesterol and lipid metabolism, inflammation, and immune response. However, their role in tumorigenesis and tumor development is still unclear. Previous studies have shown that the patterns of LXRα expression between cancer and normal tissues are diverse and depend on cancer types [12]. Therefore, we used the Oncomine database (https://www.oncomine.org/, accessed on 22 August 2021) to analyze LXRα expression over a wide range of cancers [30]. The thresholds were set as a *p*-value of 0.001, a fold change of 1.5, a gene ranking in the top 5%, and mRNA data type. The results indicated that LXRα expression was lower in breast cancer, colorectal cancer, liver cancer, lung cancer, myeloma, and sarcoma than in normal tissues. In addition, higher expression was found in esophageal cancer, kidney cancer, leukemia, and lymphoma in some datasets (Figure 2A). The details of LXRα expression in multiple cancers are summarized in Table 1.

To further evaluate LXRα expression in different human cancers, we examined LXRα expression using RNA-seq data via the TIMER 2.0 database (http://timer.cistrome.org/, accessed on 22 August 2021) [31,32]. There are over 10,897 samples across 32 cancer types on aggregate from The Cancer Genome Atlas, and we can evaluate gene expression in pan-cancer analysis. The differential LXRα expression patterns between cancer and adjacent normal tissues are shown in Figure 2B. The level of LXRα was found to be significantly lower in breast invasive carcinoma (BRCA), colon adenocarcinoma (COAD), kidney chromophobe (KICH), lung adenocarcinoma (LUAD), and lung squamous cell carcinoma (LUSC) than in adjacent normal tissues. Meanwhile, the mRNA level of LXRα was significantly higher in esophageal carcinoma (ESCA), head and neck squamous cell carcinoma (HNSC), head and neck squamous carcinoma-HPV positive (HNSC-HPV positive), kidney renal clear cell carcinoma (KIRC), and hepatocellular carcinoma (HCC) compared with adjacent normal tissues. Taking the two databases together, due to the heterogeneous data collection methods and the different biological properties of cancers, the expression levels of LXRα vary in some cancers, such as liver and kidney cancers. However, the expression of LXRα is consistent in breast, colon, and lung cancers.

**Table 1:** LXRα expression in cancerous tissue versus normal tissue in Oncomine.

Cancer Site	Cancer Type	*p*-Value	Sample Size	Fold Change	Up-/Downregulated Gene Rank	Reference
Breast	Intraductal cribriform breast adenocarcinoma	1.43 × 10^−12^	3	−1.945	112 (1%)	TCGA
Colorectal	Rectosigmoid adenocarcinoma	4.73 × 10^−5^	10	−1.732	781 (4%)	[33]
Esophageal	Esophageal adenocarcinoma	4.24 × 10^−18^	75	2.851	101 (1%)	[34]
	Barrett’s esophagus	3.11 × 10^−12^	15	5.769	120 (1%)	[34]
	Esophageal adenocarcinoma	8.15 × 10^−4^	8	2.488	345 (3%)	[35]
Kidney	Clear cell renal cell carcinoma	2.85 × 10^−6^	26	1.818	430 (3%)	[36]
	Clear cell renal cell carcinoma	1.31 × 10^−13^	23	1.625	443 (4%)	[37]
Leukemia	Acute myeloid leukemia	3.42 × 10^−55^	542	1.809	56 (1%)	[38]
	Acute myeloid leukemia	2.58 × 10^−11^	23	3.240	57 (1%)	[39]
Liver	Cirrhosis	7.34 × 10^−11^	58	−1.814	341 (3%)	[40]
	Hepatocellular carcinoma	8.79 × 10^−8^	38	−1.578	560 (5%)	[40]
	Liver cell dysplasia	5.15 × 10^−4^	17	−1.519	398 (3%)	[41]
Lung	Lung carcinoid tumor	3.78 × 10^−8^	20	−7.888	404 (5%)	[42]
Lymphoma	Centroblastic lymphoma	1.63 × 10^−24^	28	46.208	14 (1%)	[43]
	Mantle cell lymphoma	1.38 × 10^−6^	8	3.976	150 (2%)	[43]
	Diffuse large B-cell lymphoma	6.37 × 10^−8^	32	8.966	152 (2%)	[43]
	Burkitt’s lymphoma	9.81 × 10^−7^	7	8.495	368 (5%)	[43]
	Primary effusion lymphoma	1.39 × 10^−5^	9	6.261	389 (5%)	[43]
	Diffuse large B-cell lymphoma	2.19 × 10^−32^	44	15.835	42 (1%)	[44]
	Follicular lymphoma	5.77 × 10^−33^	38	9.725	55 (1%)	[44]
	Activated B-cell-like diffuse large B-cell lymphoma	1.24 × 10^−15^	17	18.080	75 (1%)	[44]
	Germinal center B-cell-like diffuse large B-cell lymphoma	3.56 × 10^−7^	9	14.761	312 (2%)	[44]
	Diffuse large B-cell lymphoma	2.78 × 10^−4^	6	3.532	71 (1%)	[45]
	Hodgkin’s lymphoma	2.33 × 10^−7^	12	5.190	155 (1%)	[46]
	Diffuse large B-cell lymphoma	3.35 × 10^−6^	11	5.417	638 (4%)	[46]
	Follicular lymphoma	3.43 × 10^−4^	5	2.201	974 (5%)	[46]
	Unspecified peripheral T-cell lymphoma	4.82 × 10^−15^	28	11.346	281 (2%)	[47]
	Angioimmunoblastic T-cell lymphoma	1.51 × 10^−6^	6	25.312	710 (4%)	[47]
Myeloma	Multiple myeloma	1.05 × 10^−6^	74	−1.931	226 (5%)	[48]
Sarcoma	Pleomorphic myxofibrosarcoma	2.97 × 10^−6^	3	−3.662	109 (1%)	[49]
	Leiomyosarcoma	3.73 × 10^−6^	26	−8.192	627 (5%)	[49]
Other	Vulvar intraepithelial neoplasia	3.55 × 10^−6^	9	−3.109	71 (1%)	[50]

## 4. Biological Functions and Mechanisms of LXRα in Diverse Cancers

Some studies have indicated that the abnormal expression of LXRs is a potential tumor-specific signature and can be involved in the clinical characteristics of malignant tissues, suggesting the indelible role of LXRs in different types of tumors [12]. LXRα might be related to tumor cell proliferation, differentiation, progression, and metastasis, and could be regarded as a potential prognostic and diagnostic biomarker in carcinogenesis.

### 4.1. Gastric Cancer 

Gastric cancer (GC) is one of the five most common cancers globally and one of the leading causes of cancer-related deaths worldwide. In addition to Helicobacter pylori infection, many recent studies have confirmed that the abnormal expression of oncogenes and tumor suppressor genes can lead to the abnormal differentiation and metabolism of GC cells [51].

Recently, using immunohistochemistry, Yu et al. found that the expression level of LXRα was significantly lower in GC tissue than in adjacent normal mucosas, and was related to the differentiation degree of cancer tissue. The lower the differentiation level of GC tissue, the lower the expression level of LXRα. They determined that decreased LXRα expression could potentially inhibit the differentiation of GC cells by activating the Wnt/β-catenin signaling, whereas the activation of LXRα had the opposite effect [52]. Whether LXRα could affect the invasion or epithelial–mesenchymal transition (EMT) ability of GC cells remains uncertain. However, different from the results of Yu et al., a study conducted by Ji et al. revealed that the mRNA and protein levels of LXRα were highly expressed in GC tissues and cell lines. Their results ultimately suggest that LXRα is an oncogene in GC and increases the expression of MMP-2/MMP-9 to promote invasion and EMT by regulating the activity of PI3k/Akt/NF-κB in GC cell lines [53]. Additionally, Zhang et al. revealed that the X-linked ectodermal dysplasia receptor, a tumor suppressor gene, could promote differentiation and inhibit the proliferation and migration of GC cells by increasing the expression of p65/LXRα to deactivate the Wnt/β-catenin pathway [54]. A recent study suggested that hypoxia can induce the expression of LXRα and promote the migration and invasion of GC cells [55]. Therefore, it is necessary to further clarify the expression level of LXRα in GC and to understand the role of LXRα in the pathological mechanism of GC to determine its potential clinical value.

### 4.2. Liver Cancer

Many factors can lead to liver cancer, such as hepatitis virus infection and liver steatosis. Recent data indicate that LXRα is correlated with multiple mechanisms to modulate the process of HCC tumorigenesis. Moriishi et al. proved that the hepatitis C virus (HCV) core protein could be degraded by the proteasome activator PA28γ and activate sterol-regulatory-element-binding protein (SREBP)-1c promoter via an LXRα/RXRα-dependent pathway, eventually accelerating the development of hepatic steatosis and HCC [56]. A subsequent study showed that LXRα was highly expressed in patients with nonalcoholic fatty liver disease and HCV infection in the liver tissues. At the same time, lipogenic targets of LXRα were also overexpressed, such as peroxisome-proliferator-activated receptor-γ (PPAR-γ), SREBP-1c, SREBP-2, and fatty acid synthase (FAS), suggesting that these genes might be involved in HCC carcinogenesis [57]. Additionally, another study suggested that HCV core protein and nonstructural protein 5A might regulate lipogenesis mediated by LXRα and contribute to liver steatosis and HCV replication [58]. Kim et al. demonstrated that HBV X protein (HBx) could interact with LXRα and facilitate the binding of LXRα to LXRE, thereby leading to the overexpression of SREBP-1c and FAS, and eventually stimulating hepatic lipid accumulation [59]. Na et al. also found these results and suggested that HBx could augment the transactivation function of LXRα by promoting CREB binding protein to interact with target gene promoters [60].

LXRα can affect the lipid metabolism of the liver and inhibit the proliferation and growth of HCC cancer cells. For example, one study indicated that the expression and activity of LXRα could be decreased by c-Fos, a component of the AP-1 transcription factor, thereby leading to changes in hepatocyte morphology, the formation of necrotic foci, and immune cell infiltration, eventually increasing proliferation, dedifferentiation, and DNA damage [61]. Previous studies have shown that the proliferation-specific regulator forkhead box M1 (FOXM1) was associated with developing HCC and upregulated the transcription of cell cycle genes, such as cyclin D1 and B1, enhancing cell cycle progression and proliferation. Hu et al. determined that LXRα could bind to the inverted repeat IR2(52-CCGTCACGTGACCT-39) region in the promoter of FOXM1 and repress the expression of FOXM1, leading to the suppression of HCC proliferation [62]. Recently, He et al. indicated that LXRα could modulate the HULC/miR-134-5p/FOXM1 axis, inhibiting HCC cell growth [63]. Moreover, another study suggested that LXRα could enhance the stability of the suppressor of cytokine signaling 3 (SOCS3) mRNA and elevate the level of SOCS3, resulting in a decline in cyclinD1 and an increase in p21 and p27, eventually leading to cell cycle arrest at the G1/S phase and inhibiting the growth of HCC cells [64].

Researchers also demonstrated that LXRα overexpression might inhibit the ability of TGFβ-induced Snail expression by deactivating Snail’s promoter, leading to the inhibition of mesenchymal differentiation, the suppression of epithelial cell proliferation, and the generation of ROS [65]. Another study showed that LXRα activation was negatively correlated with the differentiation of TGFβ-dependent cancer-associated fibroblasts by inhibiting the promoter activity of ACTA2 and limiting the growth of primary HCC [66]. Lin et al. found that LXRα activation could also increase the transcription level of miRNA-378a and enhance the potency of sorafenib in HCC [67]. In addition, recent studies have suggested that the activation of LXRα may induce the accumulation of free fatty acids in HCC cells causing lethal lipotoxicity, thereby providing an additional means for drug-resistant HCC patients [68,69]. A recent study showed that LXRα activation upregulated saturated fatty acid levels in HCC cells, while the RAF proto-oncogene serine/threonine protein kinase (Raf-1) could activate stearoyl-CoA desaturase (SCD1) to desaturate the saturated fatty acids. They then found that DFG-out Raf inhibitors could inhibit Raf-1, leading to SCD1 degradation that aroused the overload of toxic saturated fatty acids, consequently resulting in the apoptosis of HCC cells. Therefore, the combinatorial lipotoxic therapy between specific LXRα agonists and DFG-out Raf inhibitors may be exploited to develop potent cancer therapeutics [69]. Based on these studies, we conclude that LXRα is potentially a key modulator of liver cancer development and might be a potential metabolic target of liver cancer.

### 4.3. Pancreatic Cancer

Pancreatic cancer (PC) is one of the most lethal cancers, and the five-year survival rate of pancreatic ductal adenocarcinoma (PDAC) is only 5%. Yang et al. determined that the expression levels of LXRα, SREBP-1c, and polynucleotide kinase/phosphatase (PNKP) were reduced in PC cancer tissues compared with normal pancreatic tissues. They then identified that the LXRα/SREBP-1c/PNKP signaling pathway is involved in DNA repair in PC cells. Specifically, hydrophobic triptolide can bind to LXRα to inhibit the LXRα/SREBP-1c/PNKP axis, causing severe defects in DNA repair, leading to increased levels of intracellular DNA breaks, activating p53 to further induce apoptosis in PC cells beyond the apoptotic threshold, thus exerting an antitumor effect [70].

### 4.4. Colorectal Cancer

Previously, several studies elaborated that LXRα plays an essential role in lipid metabolism, modulating the transcription of genes related to the metabolism of cholesterol and fatty acids [71]. Many studies highlighted that colorectal cancer (CRC) had a very close connection with cholesterol levels; high total cholesterol (≥240 mg/dL) was positively associated with the risk of colon cancer [72,73]. Some in vivo studies illuminated that cholesterol metabolite oxysterols, as the natural ligand of LXRα, could accelerate the apoptosis of colon cancer cells and inhibit cell proliferation [74,75,76]. Some studies found that the expression of LXRα was decreased in colon cancer specimens compared with normal samples [77,78].

Uno et al. found that LXRα could bind to the central armadillo repeats (amino acid fragment 131–680) of β-catenin and inhibit its activity, leading to the decreased proliferation of colon cancer cells [79]. Another study determined that the overexpression of LXRα could block the cell cycle, induce caspase-dependent apoptosis, and impede the growth of cancer cells [77]. Vedin et al. also suggested that the activation of LXRα resulted in robust cell cycle arrest in CRC cell lines and suppressed the proliferation of cancer cells [80]. Recently, Wang et al. identified RAS protein activator-like 1 (RASAL1) as an antitumor gene that could downregulate the expression level of SCD1 and inhibit cell proliferation in CRC via the LXRα/SREBP-1c signaling pathway [81]. Based on these results, activating the expression of LXRα might be a promising treatment strategy for colon cancer.

### 4.5. Lung Cancer

Korehito Kashiwagi et al. investigated the expression of LXRα in small-cell lung carcinoma and normal lung tissue and found that it exists in alveolar macrophages and is not evident in lung epithelial cells [82]. This result suggests that LXRα might play a more significant role in affecting the function of macrophages, such as participating in the inflammatory signaling pathway. Indeed, many studies found that LXRα activation could reduce acute lung injury [83,84,85]. However, one study showed that LXRα activation could inhibit the migration and tubulogenesis of human umbilical vein endothelial cells, repressing the process of neoangiogenesis. Specifically, LXRα activation can affect endothelial cell proliferation, mainly by impairing vascular endothelial growth factor receptor 2 (VEGFR2) phosphorylation and weakening VEGFR2 compartments in lipid rafts/caveolae. Subsequently, LXRα activation was found to reduce tumor angiogenesis in Lewis lung carcinoma-1 cells’ tumor grafts [86]. Dai et al. indicated that LXRα and LXRβ double-ablated mice could spontaneously develop peripheral squamous cell lung cancer [87]. Recently, a retrospective study found that the percentage of LXRα-positive cells in stage II non-small cell lung cancer (NSCLC) patients was higher than that in stage III NSCLC patients (58% vs. 41%, *p*  = 0.04), indicating that LXRα might be an influential factor in the TNM staging system for further improving NSCLC treatment [88]. These results raise the possibility that the LXRα may be involved in the growth of lung cancer and could be a potent target for lung cancer adjunctive therapies.

### 4.6. Renal Cell Carcinoma

Clear-cell renal-cell carcinoma (ccRCC) is the most knowledgeable subtype of RCC, constituting 75% of RCC [89]. Wang et al. suggested that LXRα expression was higher in ccRCC cancer tissues than in normal tissues and was correlated with the poor prognosis of ccRCC. They then found that LXRα overexpression could inhibit the expression of NLRP3 inflammasome in ccRCC cells and promote metastasis in vivo [90]. In conclusion, LXRα may be an oncogene related to the diagnosis and prognosis of RCC and could become a novel therapeutic target.

### 4.7. Breast Cancer

Previous studies identified that LXR activation could induce the expression of estrogen deactivation enzyme and estrogen sulfotransferase, exert an indirect antiproliferative function, and inhibit the growth of breast cancer in a nude mouse model [91]. Similarly, Vedin et al. showed that the antiproliferative role of LXRs was correlated with the estrogen signaling pathway using in vitro experiments. The activation of LXRs resists estrogen-induced cell proliferation by reducing estrogen receptor α at the mRNA and protein levels, but the exact mechanism remains to be further investigated [92]. Recently, a high expression of LXRs has been identified as a biomarker for a poor prognosis [93,94,95].

Tumor immune evasion is mainly caused by the abnormal metabolism of immune cells in the tumor microenvironment. The results of single-cell RNA sequencing suggested that triple-negative breast cancer (TNBC) tumor-resident immune cells showed a high expression of LXRα, and Seurat cell-cluster analysis showed that LXRα expression was most induced in the “myeloid cell” cluster, suggesting that the upregulation of LXRα may be associated with tumor cell–immune cell interactions in TNBC. They also found that TNBC cells could produce LXRα agonists in macrophages and cytotoxic CD8+ T cells. Moreover, they illustrated that the TNBC-induced activation of LXRα could inhibit macrophage M1/M2 polarization and prohibit the normal immune function of CD8+ T cells. Therefore, they deemed that LXRα inverse agonists could be used for TNBC immunotherapies [96]. Recently, Han et al. found that LXRα was lowly expressed in breast cancer tissues and implied that it was a tumor suppressor gene that could inhibit the p56 expression of the NF-κB pathway in breast cancer cells [97]. Thus, LXRα may be a prognostic biomarker for breast cancer, and targeting LXRα might provide a novel treatment strategy.

### 4.8. Endometrial Carcinoma and Ovarian Cancer

A recent study detected the expression of LXRα between normal endometrial tissues and endometrial carcinoma (EC) tissues and found that LXRα expression was upregulated in cancer tissues, implying that LXRα might be correlated with the development of EC. They then conducted cell viability analysis and flow cytometry in Ishikawa cells. The results showed that LXRα agonist TO901317 could inhibit cell proliferation and arrest the cell cycle by inhibiting the expression of cyclin D1 and cyclin E [98]. Therefore, LXRα activation will favor the prevention of EC and may produce potential therapeutic methods.

During treatment, ovarian cancer patients with malignant ascites generally have a poor prognosis and increased resistance to multiple drugs, such as cisplatin and paclitaxel. Kim et al. indicated that enriched cholesterol in malignant ascites could activate LXRα/β to upregulate the expression of drug efflux pump proteins and contribute to chemoresistance in ovarian cancer cells [99]. This finding will help better manage and monitor the clinical application of chemotherapeutic drugs in cancer.

### 4.9. Prostate Cancer

The role of LXRs in prostate cancer (PCa) cells and animal models has been widely studied [100]. Fukuchi et al. found that the T0901317-induced activation of LXRα could interrupt the normal PCa cell cycle process and inhibit the proliferation of PCa cells by upregulating the expression of p27 [101]. With more in-depth research, an increasing number of analyses have indicated that LXRα activation could induce ABCA1 expression, which could play a role in antiproliferation activity in PCa progression [102,103,104]. Therefore, targeting LXRα may be a promising direction for prostate cancer treatment in the future. Recently, Song et al. found that SULT2B1b sulfotransferase could produce sulfated oxysterols that were identified as inhibiting LXR activation and showed that SULT2B1b expression was undetectable in the clinical samples of castration-resistant prostate cancer patients. They subsequently demonstrated that a novel LXRα/ERRα/AKR1C3/ERK1/2 survival axis could activate SULT2B1b-vanished CRPC cells. Therefore, this novel axis might be utilized in exploring novel therapeutics against CRPC [105].

### 4.10. Other Tumors

Geyeregger et al. suggested that LXR activation could suppress the interleukin-mediated proliferation of normal and leukemic T-cell blasts. Moreover, the activation of LXRα could bring about leukemic B-cell apoptosis in chronic lymphocytic leukemia (CLL) patients [106]. This result implies that targeting LXRα may be a promising therapeutic strategy for CLL patients. LXRα is also involved in glioblastoma (GBM) tumorigenesis. A study by Fang et al. showed that the overexpression of YTHDF2, mediated by the EGFR/SRC/ERK signal pathway, could expedite the m6A-dependent mRNA depletion of LXRα and HIVEP2, which facilitated cholesterol disturbances and the invasive growth of GBM [107]. Some studies have also identified that LXRα plays an essential role in osteosarcoma. Chang et al. described that LXRα activation could upregulate some of the genes relevant to the cell cycle, such as p21 and p27, mediated by the activation of FoxO1 in Saos-2 and U2OS cells [108]. These results suggest that LXRα might be a suppressor in the invasion of osteosarcoma.

**Figure 3:**
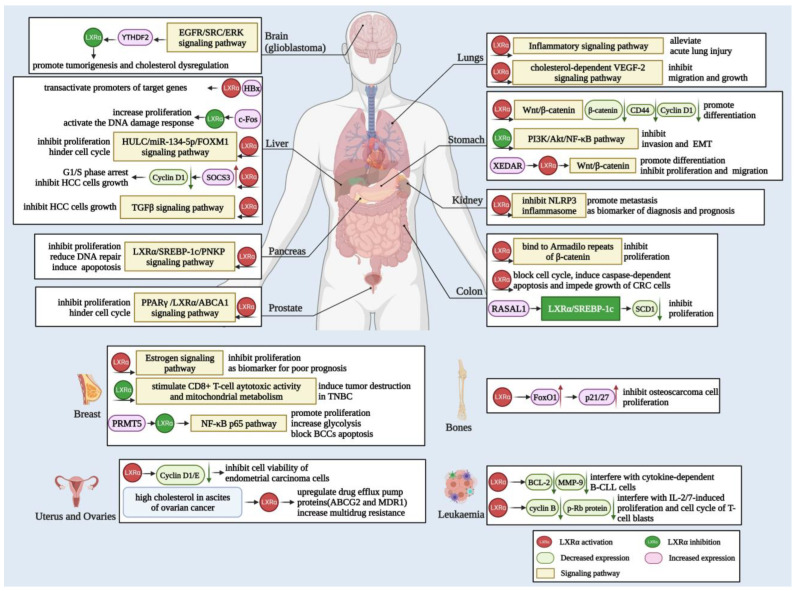
Biological function and mechanisms of LXRα in various cancers. LXRα is related to the carcinogenesis of different cancers, including glioblastoma, liver, lung, stomach, kidney, colon, pancreas, prostate, breast, and other cancers depicted in the figure. LXRα mainly exerts function in cancer cells via the Wnt/β-catenin, NF-κB, EGFR/ERK, and TGFβ signaling pathways to regulate cell differentiation, migration, invasion, EMT, and apoptosis. Moreover, the activation of LXRα also modulates target genes, such as NLRP3, PPARγ, SREBF-1c, ABCA1, and other targets, leading to proliferation inhibition, cell cycle arrest, DNA repair abnormality, and other outcomes.

Consequently, in addition to cholesterol and lipid metabolism, LXRα also plays a vital role in many types of tumors, so targeted LXRα can make a specific contribution to the development of tumor therapy. Herein, we summarize the above information in detail in Figure 3.

## 5. Therapeutic Insights into LXRα

As a member of the nuclear receptor superfamily, previous studies have suggested that targeting LXRα can be used to treat atherosclerosis and inflammation-related diseases. To date, many studies have revealed that abnormal lipid metabolism could promote the carcinogenesis, invasion, and metastasis of various cancers [109]. Due to the rapid growth and high biosynthesis of tumors, tumor cells need time to adapt to metabolic signals and dispel the overabundance of toxic metabolites. Because of the LXRα property of maintaining cholesterol and lipid homeostasis, it can be exploited for developing novel therapeutics against cancers via modulating the accumulation of metabolic products.

Moreover, studies have indicated that LXRα is correlated with the proliferation and invasion of many cancers and might be used as a potential therapeutic target in cancers. Recently, Shiragannavar et al. indicated that withaferin A, derived from the *Withania somnifera* plant, could activate the activity of LXRα, leading to the inhibition of NF-κB transcription activity, suppressing the proliferation, migration, and invasion of HCC cells. These data imply that withaferin A could be a potent anticancer compound targeting LXRα, decreasing the expression of various angiogenesis and inflammatory markers and contributing to the therapeutics of HCC [110]. One study indicated that 1, 6-O, O-diacetylbritannilactone, extracted from *Inula britannica*, could inhibit the proliferation of oral squamous cell carcinoma (OSCC) cells and impair the migration and invasion of OSCC cells, mediated by miR-1247-3p/LXRα/ABCA1 signaling [111]. Yang et al. suggested that lycopene could inhibit the proliferation of androgen-dependent human prostate tumor cells via the activation of the PPARγ/LXRα/ABCA1 signaling pathway [104]. 

In addition to the previously discovered LXR classic ligands, increasingly more natural or synthesized small molecular compounds have been found to specifically regulate LXRα activation. Several potential therapeutic agents for some cancers are shown in Table 2. Although the therapeutic methods targeting LXRα are not yet fully developed, more therapeutic achievements could be attained in the future due to the knowledge regarding the mechanisms and functions of LXRα in cancer. 

## 6. Conclusions

In this review, we summarized the potential mechanisms of LXRα that modulate multiple processes in different cancers. Because of the cancer-specific expression characteristic of LXRα, it can exert contrary functions in tumor inhibition and promotion. The expression of LXRα in most types of cancer cells is decreased (e.g., lung, breast, and kidney cancer cells), and its overexpression can suppress the EMT, metastasis, and proliferation of cancer cells; however, it can exert a contentious effect in some cancers, such as gastric cancer and liver cancer. This phenomenon may be caused by the heterogeneity of the included clinical samples in different studies, the various activated ligands of LXRα, or the different signaling pathways regulated by LXRα. However, most studies have found that the activation of LXRα can inhibit cancer pathogenesis. Although some studies have emphasized the relationship between LXRα expression and patient prognosis (e.g., breast, kidney, and prostate cancer), further understanding of the diagnostic value of LXRα is needed.

Even though numerous studies have demonstrated that LXRα expression decreases in cancers and may serve as a novel therapeutic target, its clinical application remains challenging. At present, most of the activating ligands for LXRα are non-specific and target LXRα and LXRβ at the same time; thus, more natural small-molecule substances and manual interventions that specifically target LXRα should be studied. Moreover, the excessive activation of LXRα may also affect the normal function of the cholesterol and lipid metabolism signaling pathways, which may cause unnecessary side effects. Therefore, how to specifically target LXRα against cancer without severe cholesterol metabolism abnormalities is worth considering in future investigations. In short, LXRα plays a vital role in the occurrence and development of cancer, and it could be a target for adjuvant cancer treatment. However, some problems remain to be solved, and developing more specific and effective drugs is also an urgent priority.

## Figures and Tables

**Table 2:** Potential reagents to modulate LXRα activity in cancer therapy.

Reagents	Tumor	Mechanism and Effects	Reference
1, 6-O, O-Diacetylbritannilactone	Oral squamous cell carcinoma	Activate miR-1247-3p/LXRα/ABCA1 signaling to inhibit progression and promote apoptosis of cancer cells	[111]
Withaferin A	Hepatocellular carcinoma	Activate LXRα to inhibit the transcriptional activity of NF-κB and suppress the proliferation, migration, invasion, and anchorage-independent growth of HCC cells	[110]
GW3965	Sorafenib-resisted HCC	LXRα activation increases the expression of miRNA-378a-3p, which can induce the apoptosis of sorafenib-resistant HCC cells	[67]
GW3965	Colorectal cancer	LXRα activation decreases the expression of EGFR and suppresses the proliferation of colorectal cancer cells	[62]
Lycopene	Prostate cancer	Activates the PPARγ/LXRα/ABCA1 pathway and inhibits the proliferation of cancer cells	[104]
GW3965	Prostate cancer	Activates LXRα to inhibit the growth and metastasis of prostate cancer cells via the EGFR/AKT/FOXO3A pathway	[112]
Celastrol	Clear-cell renal-cell carcinoma	Activation of LXRα promotes ABCA1-mediated cholesterol efflux and inhibits EMT, ultimately suppressing cancer cell proliferation, migration, invasion, and tumor growth	[113]

## Data Availability

Not applicable.
